# Role of Kras Status in Patients with Metastatic Colorectal Cancer Receiving First-Line Chemotherapy plus Bevacizumab: A TTD Group Cooperative Study

**DOI:** 10.1371/journal.pone.0047345

**Published:** 2012-10-12

**Authors:** Eduardo Díaz-Rubio, Auxiliadora Gómez-España, Bartomeu Massutí, Javier Sastre, Margarita Reboredo, José Luis Manzano, Fernando Rivera, MªJosé Safont, Clara Montagut, Encarnación González, Manuel Benavides, Eugenio Marcuello, Andrés Cervantes, Purificación Martínez de Prado, Carlos Fernández-Martos, Antonio Arrivi, Inmaculada Bando, Enrique Aranda, Spanish Cooperative Group for the Treatment of Digestive Tumors (TTD), E. Aranda, A. Gómez, B. Massutí, A. Yuste, E. Díaz Rubio, J. Sastre, M. Valladares, A. Abad, F. Rivera, MªJosé Safont, M. Gallén, E. González, M. Benavides, E. Marcuello, M. Tobeña, A. Cervantes, P. Martínez de Prado, C. Fernández-Martos, A. Arrivi, A. López-Ladrón, A. Lacasta, M. Llanos, J. Remón, A. Anton, J. Mª. Vicent, A. Gala´n, R. Dueñas, J. Mª. Tabernero, H. Manzano, Mª. J. Gómez, J. Alfaro, F. Losa, P. Escudero, T. García, J. L. García López, Mª L. García de Paredes, A. Velasco, D. Almenar, R. Vera, J. L. García Puche, A. Carrato, A. Rodriguez Lescure, E. Jiménez, V. Alberola, J. García-Foncillas, M. Constenla, A. Ruiz, P. Bueso, E. Cabrera, L. del Río,, J. Ponce, A. Oltra, T. Checa, A. Etxeberría, C. Alonso

**Affiliations:** H. Reina Sofía; H. General Universitario de Alicante; H. Universitario Clínico San Carlos; C. H. Universitario La Coruña; ICO. H. Germans Trias i Pujol; H. Marqués de Valdecilla; H. General Universitario de Valencia; H. del Mar; H. Virgen de las Nieves; H. Universitario Carlos Haya; H. Santa Creu i Sant Pau; H. Cl?´nico de Valencia; H. de Basurto; Instituto Valenciano de Oncología; F. H. Son Llatzer; H. Nuestra Señora de Valme; H. de Donostia; H. Universitario de Canarias; H. de Mataró; H. Miguel Servet; H. de Sagunto; H. Ciudad de Jaén; H. Universitari Vall d'Hebrón; H. Son Dureta; H. Puerta del Mar; C. Sanitari de Terrasa; H. General de L'Hospitalet; H. C. Universitario Lozano Blesa; H. Morales Meseguer; H. Ramón y Cajal; H. Universitario de la Princesa; H. Dr. Peset; H. de Navarra; H. Clínico San Cecilio; H. General Universitario de Elche; H. Jerez de la Frontera; H. Arnau de Vilanova; C. Universitaria de Navarra; Complejo Hospitalario Pontevedra; H. de Fuenlabrada; H. de Barbastro; H. Virgen de los Lirios; I. de Oncología Coracha´n; Instituto Oncológico; H. General de Albacete; 1 Department of Medical Oncology, Hospital Clínico San Carlos (HCSC), Red Temática de Investigación Cooperativa en Cáncer (RD06/0020/0021), Facultad de Medicina, Universidad Complutense, Instituto de Investigación Sanitaria del HCSC (IdISSC), Madrid, Spain; 2 Department of Medical Oncology, Hospital Reina Sofía, Córdoba, Spain; 3 Department of Medical Oncology, Hospital General, Alicante, Spain; 4 Department of Medical Oncology, Complejo Hospitalario Universitario La Coruña, La Coruña, Spain; 5 Department of Medical Oncology, Instituto Catalán de Oncología, Hospital Germans Trias I Pujol, Badalona, Spain; 6 Department of Medical Oncology, Hospital Marqués de Valdecilla, Santander, Spain; 7 Department of Medical Oncology, Hospital General de Valencia, Valencia, Spain; 8 Department of Medical Oncology, Hospital del Mar, Barcelona, Spain; 9 Department of Medical Oncology, Hospital Virgen de las Nieves, Granada, Spain; 10 Department of Medical Oncology, Hospital Universitario Carlos Haya, Málaga, Spain; 11 Department of Medical Oncology, Hospital Santa Creu i Sant Pau, Barcelona, Spain; 12 Department of Medical Oncology, Institute of Health Research INCLIVA. University of Valencia, Valencia, Spain; 13 Department of Medical Oncology, Hospital de Basurto, Vizcaya, Spain; 14 Department of Medical Oncology, Instituto Valenciano de Oncología, Valencia, Spain; 15 Department of Medical Oncology, Fundación Hospital Son Llatzer, Palma de Mallorca, Spain; University of California Irvine, United States of America

## Abstract

**Background:**

In the MACRO study, patients with metastatic colorectal cancer (mCRC) were randomised to first-line treatment with 6 cycles of capecitabine and oxaliplatin (XELOX) plus bevacizumab followed by either single-agent bevacizumab or XELOX plus bevacizumab until disease progression. An additional retrospective analysis was performed to define the prognostic value of tumour KRAS status on progression-free survival (PFS), overall survival (OS) and response rates.

**Methodology/Principal Findings:**

KRAS data (tumour KRAS status and type of mutation) were collected by questionnaire from participating centres that performed KRAS analyses. These data were then cross-referenced with efficacy data for relevant patients in the MACRO study database. KRAS status was analysed in 394 of the 480 patients (82.1%) in the MACRO study. Wild-type (WT) KRAS tumours were found in 219 patients (56%) and mutant (MT) KRAS in 175 patients (44%). Median PFS was 10.9 months for patients with WT KRAS and 9.4 months for patients with MT KRAS tumours (p = 0.0038; HR: 1.40; 95% CI:1.12–1.77). The difference in OS was also significant: 26.7 months versus 18.0 months for WT versus MT KRAS, respectively (p = 0.0002; HR: 1.55; 95% CI: 1.23–1.96). Univariate and multivariate analyses showed that KRAS was an independent variable for both PFS and OS. Responses were observed in 126 patients (57.5%) with WT KRAS tumours and 76 patients (43.4%) with MT KRAS tumours (p = 0.0054; OR: 1.77; 95% CI: 1.18–2.64).

**Conclusions/Significance:**

This analysis of the MACRO study suggests a prognostic role for tumour KRAS status in patients with mCRC treated with XELOX plus bevacizumab. For both PFS and OS, KRAS status was an independent factor in univariate and multivariate analyses.

## Introduction

At present, standard first-line treatment for patients with metastatic colorectal cancer (mCRC) includes combination chemotherapy in conjunction with either an anti-epidermal growth factor receptor (EGFR) agent such as cetuximab [1], [2] or panitumumab [3], or an antiangiogenic agent, such as bevacizumab [4]–[6]. One critical issue is the selection of patients who will benefit from treatment with these biological agents. In the case of anti-EGFR therapies, the presence of a KRAS mutation is a negative predictive factor for response to treatment [7]–[9] and determination of KRAS status is now required by American and European authorities before these agents can be administered [10]–[13].

The prognostic value of tumour KRAS status has been extensively evaluated in patients with advanced and localised CRC, although results have been conflicting. Some studies have demonstrated a prognostic effect [14]–[19], while others have failed to show any significant prognostic effect [20]–[24].

Recent studies of chemotherapy regimens, with or without cetuximab, in the first-line treatment of patients with mCRC have sparked new interest in this issue [7], [25]–[27].

The interaction of EGFR and vascular endothelial growth factor (VEGF) is well known [28], [29], although the potential role of KRAS mutation status in patients undergoing treatment with bevacizumab remains of great interest. Retrospective analyses have shown that bevacizumab in combination with irinotecan/5-fluorouracil (5-FU)/leucovorin chemotherapy provides a significant clinical benefit for patients with mutant (MT) and wild-type (WT) KRAS tumours [30], [31]. The authors also noted that the benefit of treatment was greater in patients with WT compared with MT KRAS tumours. Other studies have shown no prognostic effect of tumour KRAS status on survival in patients receiving combination chemotherapy with bevacizumab [32]–[35].

We undertook an analysis of data from the MACRO study to evaluate the prognostic value of tumour KRAS status in patients receiving combination therapy with capecitabine plus oxaliplatin (XELOX) and bevacizumab. Correlations between KRAS status and response rate, progression-free survival (PFS) and overall survival (OS) were analysed.

## Methods

### Ethics Statement

The Institutional Review Board and Ethic Committee of Hospital Clinico San Carlos, Madrid as Reference Ethics Committee, as well as the Spanish Medicine Agency, approved the study protocol (Study TTD-05-02; EudraCT: 2005-003325-67; clinicaltrials.gov identifier NCT00335595). Study procedures were carried out in accordance with the Declaration of Helsinki and its subsequent amendments and Good Clinical Practice guidelines. Written informed consent was obtained from all patients before enrolment.

### Patients and Study Design

The design of the MACRO study has been reported previously [36]. In brief, patients aged≥18 years, with histologically confirmed mCRC, Eastern Cooperative Oncology Group performance status (ECOG PS)≤2, measurable disease, no previous chemotherapy for advanced disease, adequate hepatic and renal function, and no contraindications to bevacizumab therapy were included.

The primary endpoint of the MACRO study was PFS; secondary endpoints included OS, objective response rate (ORR), toxicity and several translational research assessments. Between July 2006 and September 2008, 480 patients were entered into the study; 239 were randomized to maintenance XELOX plus bevacizumab after induction XELOX plus bevacizumab and 241 were randomized to single-agent bevacizumab after induction XELOX plus bevacizumab. Induction XELOX consisted of 6 cycles of bevacizumab (7.5 mg/kg intravenously [iv] d1), capecitabine (1000 mg/m^2^ orally bid d1–14) and oxaliplatin (130 mg/m^2^ iv d1) every 3 weeks followed by XELOX plus bevacizumab or bevacizumab alone until progression.

### KRAS Mutation Analysis

Evaluation of KRAS status was performed retrospectively. As KRAS analysis is standard practice in Spain, sample analysis was performed either at the treating centre or centrally using existing platforms. Participating centres sent KRAS findings to the data collection centre. Data were obtained on KRAS status (WT or MT), the type of mutation found (12 Ala, 12 Arg, 12 Asp, 12 Cys, 12 Ser, 12 Val or 13 Asp) and the methodology used (method DxS, StripAssay and sequencing). These findings were correlated with patient's existing data, including response rate, PFS, OS, rescue surgery and second-line therapy.

### Statistical Analysis

The primary objective of this ancillary analysis of the MACRO study was to evaluate the utility of tumour KRAS status as a prognostic factor in patients with mCRC who were treated with chemotherapy and bevacizumab. The MACRO study demonstrated that single-agent bevacizumab was not statistically significantly inferior to XELOX plus bevacizumab as maintenance therapy [36]; therefore tumour KRAS mutation data from patients in the two treatment groups were combined for the purposes of the present analysis. PFS and OS curves were calculated according to KRAS tumour mutation status using the Kaplan–Meier method. The prognostic value of the biological marker was determined using the log-rank test. Univariate and multivariate Cox proportional hazards models were built with the following variables: ECOG PS 0–1 versus 2; age <70 versus ≥70 years; number of metastatic sites 1 versus ≥2; lactate dehydrogenase (LDH) high levels versus within the normal range; alkaline phosphatase high levels versus within the normal range; male versus female sex; KRAS MT versus WT status; maintenance treatment (XELOX–bev vs bev), prior adjuvant chemotherapy and radiotherapy and surgical removal of metastases prior to the study entry.

## Results

### Patient Characteristics

**Figure 1:**
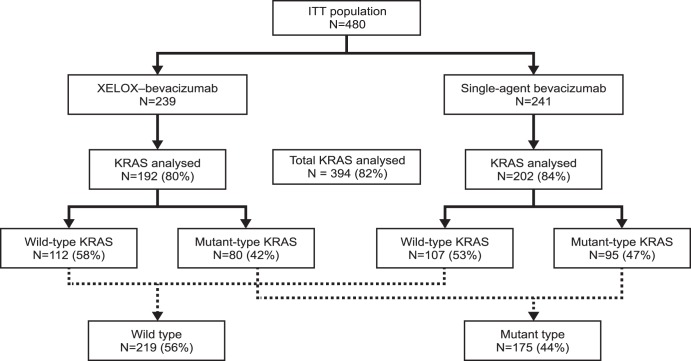
Patient flow.

The intent-to-treat (ITT) population of the MACRO study comprised 480 patients, 394 (82.1%) of whom had information on KRAS status and were included in this biomarker sub-study (Figure 1).

Patient characteristics according to KRAS status are shown in Table 1. Significant differences were observed between the two groups in prior adjuvant chemotherapy, prior radiotherapy and surgical removal of metastases prior to entry into the study.

**Table 1:** Baseline characteristics of patients included in the KRAS analysis according to tumour KRAS status (n = 394).

Characteristic	WT KRAS (n = 219)	MT KRAS (n = 175)	p-value
Median age, years (range)	63 (40–82)	64 (30–80)	
Sex, %			
Male	64.8	62.3	
Female	35.2	37.7	
ECOG PS, %			
0	61.5	50.6	
1	37.2	47.1	
2	1.4	2.3	
Primary tumour location, %			
Colon	28.3	23.4	
Rectum	60.3	64.0	
Both	11.4	12.6	
Metastases, %			
Liver only	40.2	31.4	
Locoregional	16.4	16.6	
Lung	39.3	42.9	
Other	27.4	29.7	
Prior adjuvant therapy, %			
Chemotherapy	11.4	20.0	<0.05^a^
Radiotherapy	5.5	11.4	<0.05^a^
Median no. of organs affected (range)	2 (1–5)	2 (1–6)	
Median no. of metastatic sites (range)	4 (1–20)	3 (1–11)	
Resection of primary tumour, %	71.2	78.3	
Surgery for metastatic disease prior to study entry, %	5.5	11.4	<0.05^b^
Median LDH, U/L (range)	423 (150–5386)	393.5 (95.4–5313)	
Median CEA, ng/mL (range)	36.7 (0.5–14280)	42.1 (0.8–8527)	

Abbreviations: CEA, carcinoembryonic antigen LDH, lactate dehydrogenase; ECOG PS, Eastern Cooperative Oncology Group performance status; MT, mutant; WT, wild type.

a Chi-Square Test.

b Fisher’s Exact Test.

### KRAS Analysis

Questionnaires were completed by 171 (43.4%) of Spanish reference centres and 185 (47.0%) of other centres participating in the study. The most common technique for KRAS determination was DxS (76.4% of cases), followed by sequencing (13.3%), StripAssay (10.0%) and pyrosequencing (0.3%). In total, 219 of the 394 patients (55.6%) had WT KRAS tumours, while 175 patients (44.4%) had some type of mutation. The most frequent mutations were G12D (33.3%), G12V (26.9%), G13D (21.8%), G12C (8.3%), G12S (1.9%), G12A (3.9%) and G12R (2.6%). This information was not available in 19 (10.9%) cases.

### Prognostic Value of KRAS

**Figure 2:**
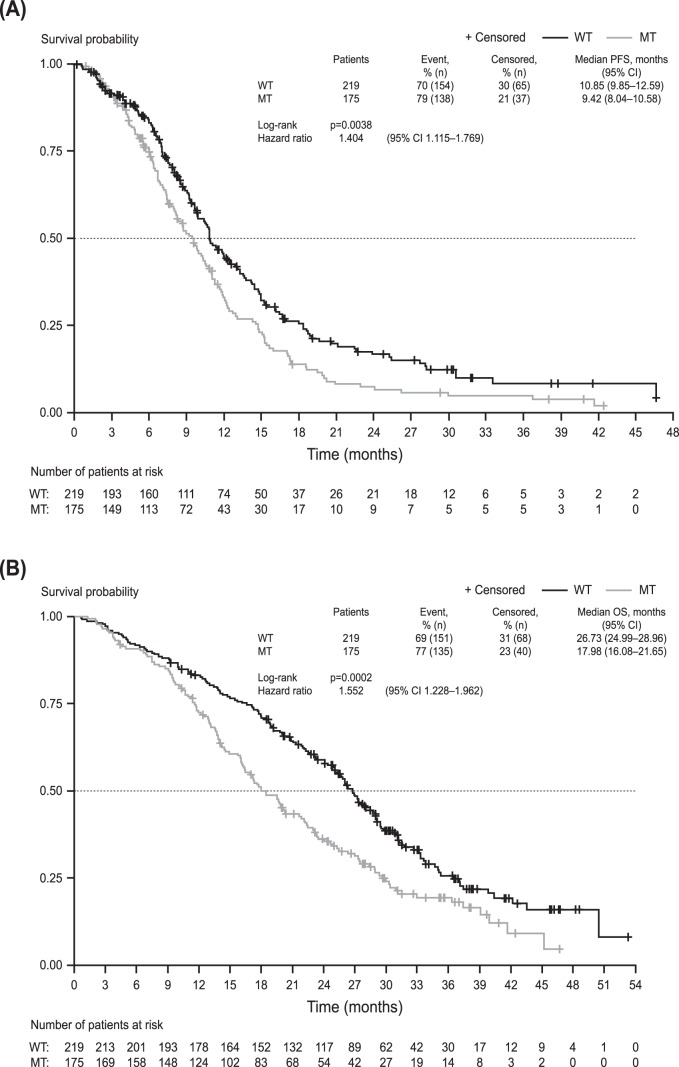
Progression-free survival (A) and overall survival (B) according to KRAS status.

The confirmed ORR was 57.5% in patients with WT KRAS tumours compared with 43.4% in patients with MT KRAS tumours (p = 0.0054; OR: 1.77, 95% CI 1.18–2.64). Median PFS was significantly longer in patients with WT versus MT KRAS tumours, 10.9 months versus 9.4 months (p = 0.0038; HR: 1.40; 95% CI: 1.12–1.77) (Figure 2A). A statistically significant difference was observed in OS (Figure 2B): patients with WT KRAS tumours had a median OS of 26.7 months versus 18.0 months for patients with MT KRAS tumours (p = 0.0002; HR: 1.55; 95% CI: 1.23–1.96).

When patients were analysed for PFS according to treatment received, a similar pattern was observed in the KRAS MT and WT groups. In the XELOX plus bevacizumab maintenance group, median PFS was 12.6 months versus 10.0 months in patients with WT and MT KRAS tumours, respectively (p = 0.0560; HR: 1.39; 95% CI: 0.99–1.95). In the single-agent bevacizumab group, median PFS was 10.8 versus 8.7 months in patients with WT and MT KRAS tumours, respectively (p = 0.0492; HR: 1.38; 95% CI, 1.00–1.89).

A total of 47 patients (11.9%) underwent salvage surgery of metastases, 28 of whom had WT KRAS tumours and 19 had MT KRAS tumours (p = 0.5574). Resection was complete (R0) in 20 patients (71.4%) with WT KRAS and 12 (63.2%) with MT KRAS tumours (p = 0.0769). Median PFS in patients who underwent salvage surgery of metastases was 16.2 months versus 12.7 months in WT and MT KRAS patients, respectively (HR: 1.91; 95% CI: 0.86–4.22; p = 0.1048). Median OS was not reached in the KRAS WT group and was 23.3 months in the MT KRAS group.

The influence of second and further lines of treatment, in particular the administration of an anti-EGFR agent after the discontinuation of the treatment of the study, was examined. Over all subsequent lines of therapy, 54 (24.7%) of the 219 patients with WT KRAS tumours had no subsequent therapy, 109 patients (49.8%) received anti-EGFR therapy, and 56 patients (25.6%) received no anti-EGFR therapy. Among the 175 patients with MT KRAS tumours, 37 (21.1%) received no further treatment, 23 (13.1%). received anti-EGFR therapy, 55 (31.4%) received chemotherapy alone and 60 (34.3%) received chemotherapy with bevacizumab.

Subsequent therapy just after the study discontinuation is described in more detail in Table 2.

**Table 2:** Subsequent therapy according to tumour KRAS status.

Regimen, n (%)	WT KRAS (n = 219)	MT KRAS (n = 175)
Anti-EGFR alone	1 (<1)	1 (<1)
Anti-EGFR + irinotecan-based chemotherapy	39 (17.8)	7 (4.0)
Anti-EGRF + capecitabine- or 5-FU-based chemotherapy	1 (<1)	
Anti-EGFR + oxaliplatin based chemotherapy	2 (0.9)	1 (<1)
Bevacizumab alone^a^	7 (3.2)	1 (<1)
Bevacizumab + irinotecan-based chemotherapy^b^	16 (7.3)	27 (15.4)
Bevacizumab + capecitabine- or 5-FU-based chemotherapy	10 (4.6)	9 (5.1)
Bevacizumab + oxaliplatin-based chemotherapy	19 (8.7)	17 (9.7)
Bevacizumab + anti EGFR + irinotecan	1 (<1)	
Irinotecan alone or irinotecan-based chemotherapy	42 (19.2)	55 (31.4)
Capecitabine or 5-FU alone or oxaliplatin-based chemotherapy^c^	27 (12.3)	20 (11.4)
No treatment	54 (24.7)	37 (21.1)

Abbreviation: EGFR: epidermal growth factor receptor.

a Patient discontinuated chemotherapy and continuous bevacizumab after the study withdrawal.

b One patient received additional gemcitabine.

c Includes one patient who received methothretaxe.

**Figure 3:**
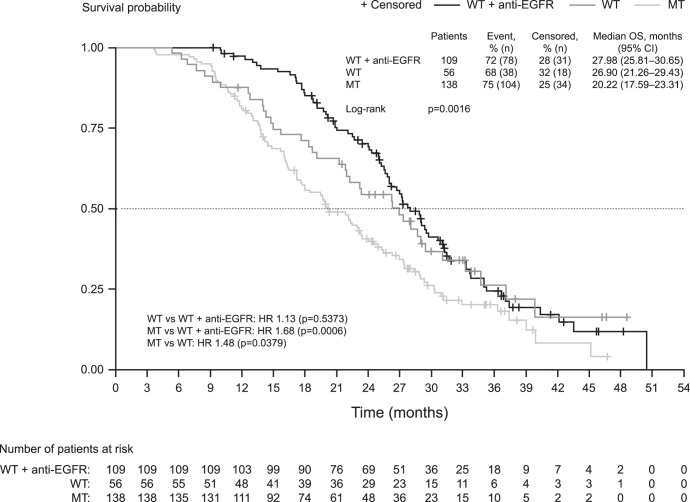
Effect of post-progression anti-EGFR therapy on survival. Abbreviations: EGFR, epidermal growth factor receptor; HR, hazard ratio; MT, mutant; WT, wild type.

Median OS was 28.0 months in patients with WT KRAS tumours who received post-study anti-EGFR therapy versus 20.2 months in those with MT KRAS tumours (HR: 1.68; 95% CI: 1.25–2.26; p = 0.0006). OS was longer in patients with WT KRAS tumours who did not receive anti-EGFR therapy than in patients with MT KRAS tumours (26.9 months versus 20.2 months; HR: 1.48; 95% CI: 1.02–2.16; p = 0.0379). There was no difference in OS between patients with WT KRAS tumours who did not receive anti-EGFR therapy and those who did (28.0 versus 26.9 months; HR: 1.13, 95% CI: 0.77–1.67; p = 0.5373) (Figure 3).

Reasons for withdrawal from the study were similar in patients with WT and MT KRAS. The most common reasons for withdrawal were: disease progression (WT KRAS n = 104 [48.4%]; MT KRAS n = 102 [58.6%]), toxicity, adverse events or intercurrent disease (WT KRAS n = 52 [24.2%]; MT KRAS n = 39 [22.4%]), surgery (WT KRAS n = 36 [16.7%]; MT KRAS n = 17 [9.8%]) and death (WT KRAS n = 6 [2.8%]; MT KRAS n = 5 [2.9%]). There were no statistically significant differences between the two groups in this respect (p = 0.1815).

### Univariate and Multivariate Analysis

Results of the univariate and multivariate analyses for PFS are shown in Table 3. For the univariate analysis, variables independently associated with PFS were: age (HR: 1.32; 95% CI: 1.02–1.70; p = 0.032); LDH level (HR: 2.02; 95% CI: 1.57–2.60; p<0.0001), alkaline phosphatase level (HR: 1.30; 95% CI: 1.03–1.64; p = 0.0264), KRAS status (HR: 1.40; 95% CI: 1.12–1.77; p = 0.0040) and surgical removal of metastases prior to the study entry (HR: 1.61; 95% CI: 1.01–2.57; p = 0.0457). In the multivariate analysis, significant predictors of PFS were: age (HR: 1.34; 95% CI: 1.01–1.76; p = 0.0422), the number of organs involved (HR:1.39; 95% CI: 1.07–1.81; p = 0.0142), LDH level (HR:2.24; 95% CI:1.68–3.01; p<0.0001), KRAS status (HR:1.47; 95% CI: 1.14–1.91; p = 0.0031) and surgical removal of metastases prior to the study entry (HR: 1.75; 95% CI: 1.04–2.94; p = 0.0367).

**Table 3:** Univariate and multivariate analyses for progression-free survival.

	Univariate analysis			Multivariate analysis		
Parameter	p-value	HR	95% CI	p-value	HR	95% CI
ECOG PS (2 vs 0–1)	0.1773	1.748	0.777–3.932	0.7731	1.144	0.458–2.861
Age (≥70 vs <70 years)	0.0320	1.318	1.024–1.696	0.0422	1.335	1.010–1.763
No. affected organs (≥2 vs 1)	0.1908	1.168	0.926–1.474	0.0142	1.392	1.069–1.814
LDH (elevated vs normal)	<0.0001	2.018	1.566–2.600	<0.0001	2.244	1.675–3.007
AP (elevated vs normal)	0.0264	1.300	1.031–1.639	0.4705	0.901	0.680–1.195
Sex (female vs male)	0.6592	1.056	0.829–1.346	0.9184	1.014	0.775–1.326
KRAS status (MT vs WT)	0.0040	1.404	1.115–1.769	0.0031	1.473	1.139–1.905
Maintenance treatment (XELOX–bev vs bev)	0.3017	1.129	0.896–1.423	0.4513	1.104	0.854–1.428
Prior chemotherapy (no vs yes )	0.7756	0.956	0.703–1.301	0.4418	0.845	0.550–1.299
Prior radiotherapy (no vs yes )	0.7988	0.951	0.643–1.404	0.5954	1.159	0.672–2.000
Surgical removal of metastases prior to the study entry(no vs yes )	0.0457	1.610	1.009–2.568	0.0367	1.746	1.035–2.944

Abbreviations: AP, alkaline phosphatase; bev, bevacizumab; CI, confidence interval; ECOG PS, Eastern Cooperative Oncology Group performance status; HR, hazard ratio; LDH, lactate dehydrogenase; MT, mutant type; WT, wild type; XELOX, capecitabine + oxaliplatin.

Predictors of OS in the univariate analysis were: number of organs involved (HR: 1.45; 95% CI: 1.14–1.83; p = 0.0023), LDH level (HR: 2.13; 95% CI: 1.65–2.75; p<0.0001), alkaline phosphatase level (HR: 1.47; 95% CI: 1.16–1.86; p = 0.0012) and KRAS status (HR: 1.55; 95% CI: 1.23–1.96; p = 0.0002). Significant factors in the multivariate analysis were the number of organs involved (HR: 1.58, 95% CI:1.22–2.06; p = 0.0006); LDH (HR: 2.27; 95% CI: 1.71–3.01; p<0.0001) and KRAS status (HR: 1.60; 95% CI: 1.24–2.08; p = 0.0004) (Table 4).

**Table 4:** Univariate and multivariate analyses of overall survival.

	Univariate analysis			Multivariate analysis		
Parameter	p-value	HR	95% CI	p-value	HR	95% CI
ECOG PS (2 vs 0–1)	0.3518	1.469	0.654–3.302	0.8815	0.933	0.376–2.314
Age (≥70 vs <70)	0.1682	1.198	0.927–1.548	0.3342	1.150	0.866–1.526
No. of affected organs (≥2 vs 1)	0.0023	1.445	1.140–1.831	0.0006	1.584	1.217–2.062
LDH (abnormal vs normal)	<0.0001	2.130	1.647–2.754	<.0001	2.266	1.706–3.011
AP (abnormal vs normal)	0.0012	1.472	1.164–1.861	0.7607	1.044	0.792–1.377
Sex (female vs male)	0.7310	0.958	0.752–1.221	0.3318	0.875	0.668–1.146
KRAS status (MT vs WT)	0.0002	1.552	1.228–1.962	0.0004	1.604	1.236–2.083
Maintenance treatment (XELOX–bev vs bev)	0.3503	1.117	0.886–1.409	0.2184	1.175	0.909–1.518
Prior chemotherapy (no vs yes )	0.1665	1.266	0.906–1.769	0.3616	1.244	0.778–1.989
Prior radiotherapy (no vs yes )	0.5676	1.135	0.735–1.755	0.9887	1.004	0.542–1.861
Surgical removal of metastases priorto study entry (no vs yes )	0.6795	1.098	0.704–1.714	0.6656	1.118	0.674–1.855

Abbreviations: AP, alkaline phosphatase; bev, bevacizumab; CI, confidence interval; ECOG PS, Eastern Cooperative Oncology Group performance status; HR, hazard ratio; LDH, lactate dehydrogenase; MT, mutant type; WT, wild type; XELOX, capecitabine + oxaliplatin.

## Discussion

The present analysis of the MACRO study indicates that tumour KRAS status is a prognostic factor in patients with mCRC receiving bevacizumab in combination with capecitabine plus oxaliplatin. Patients with WT KRAS tumours had a significantly greater clinical benefit than those with MT KRAS tumours in terms of ORR (57.5% versus 43.4%), PFS (10.9 versus 9.4 months) and OS (26.7 versus 18.0 months). The same trend was observed when both treatment arms were combined and when analysed separately. Univariate and multivariate analyses showed the independent role of KRAS status for both PFS and OS. This indicates that in the MACRO study, KRAS was a prognostic factor in patients with mCRC receiving bevacizumab in combination with chemotherapy.

The prognostic value of KRAS was initially suggested by the RASCAL I study, which included 2721 patients [16]. Multivariate analysis established that the effect of KRAS was independent of other variable factors such as sex, tumour site or Dukes stage. The RASCAL study suggested that KRAS was important not only for carcinogenesis of CRC but also for prognosis in patients with all stages of the disease. Other studies have confirmed the prognostic value of KRAS status [14], [15], [17]–[19], [25], [26].

Several studies have evaluated the role of KRAS status in patients with mCRC receiving first-line treatment with bevacizumab (Table 5) [30]–[35]. In the study by Ince et al, no statistically significant difference was observed for OS in KRAS WT and MT bevacizumab-treated patients (27.7 versus 19.9 months for WT and MT KRAS, respectively; HR: 0.64; 95% CI 0.35–1.15) [30]. In the subsequent analysis by Hurwitz et al., response rates (60% versus 43%) and PFS (13.5 versus 9.3 months) were numerically greater for bevacizumab-treated patients with WT versus MT KRAS tumours, although the difference in PFS was not statistically significant (HR 0.66; p = 0.09) [31]. Other studies have also reported that KRAS mutation status is not a prognostic factor for patient outcome [32]–[35]. This variability in results could be a result of several factors, including the number of patients included in the different trials, the proportions of patients tested for KRAS mutation status and the technology used, the chemotherapy regime under investigation, and subsequent second- and third-line therapies.

**Table 5:** Summary of KRAS data from larger studies of bevacizumab + chemotherapy in patients with metastatic colorectal cancer.

	Hurwitz (31)	CAIRO2 (35)	AGITG MAX (33)	PACCE (34)	MACRO
Regimen	IFL + Bev	Cape + Ox + Bev	Cape+Bev± MitC	CT (Iri, Ox) + Bev	Cape + Ox + Bev
No of patients tested/No. of patients in study	129/402	264/368	212/314	425/525	394/480
Patients with KRAS mutation, %	34	41	29	40	44
Availability for KRAS analysis, %	32	72	67	82	82
Response rate, %					
WT	60	50	41–45	48–56	58
MT	43	59	24–46%	38–44	43
	p = NA	P = 0.16	p = NS	p = NS	p = 0.0054
PFS, months					
WT	13.5	10.6	8.8	11.5–12.5	10.9
MT	9.3	12.5	8.2	11.0–11.9	9.4
	p = 0.09	P = 0.80	p = NS	p = NA	p = 0.0038
OS, months					
WT	27.7	22.4	19.8	19.8–24.5	26.7
MT	19.9	24.9	17.6	19.3–20.5	18
	p = NA	p = 0.82	p = NS	p = NA	p = 0.0002

**Abbreviations:** bev, bevacizumab; Cape, capecitabine; CT, chemotherapy; Iri, irinotecan; MitC, mitomycin C, MT, mutant type; NA, not available; NS, not significant; Ox, oxaliplatin; WT, wild type.

Our study did not aim to determine whether KRAS status was predictive in patients receiving bevacizumab, as all patients were treated with bevacizumab. Results from the studies by Hurwitz et al. [31] and Ince et al. [30] appear to show that KRAS status is not predictive and that all patients can benefit from bevacizumab treatment. Our study did not include a bevacizumab-free control arm and therefore we cannot state with certainty that patients with MT KRAS tumours benefit from bevacizumab treatment; however, bevacizumab does not seem to have an adverse effect on survival, as has been seen in some studies of the anti-EGFR agents [3], [35]. The benefit of treatment with bevacizumab appears to be greater in patients with WT KRAS tumours.

Our study has several limitations. Firstly, the KRAS mutation analysis was retrospective, which is common in randomized studies examining the role of biomarkers [7], [8], [31]. Secondly, KRAS mutation analyses were performed either at the treatment centre or at a Spanish reference centre, with the result that different technologies were used, potentially introducing a bias. However, the percentage and type of mutations found in MACRO was consistent with reports from other studies [7], [8], as well as the Determina KRAS project, which has analysed samples from 12 262 patients in five Spanish reference centres [37]. In addition, KRAS data were not available for 18% of the population, potentially introducing selection bias. Another possible bias in our study is the imbalance observed in some parameters between patients with WT and MT KRAS tumours (prior adjuvant therapy and surgery for metastatic disease prior to the study entry). Whether this is a chance finding, or because patients with MT KRAS tumours have a different natural history with more aggressive disease, remains to be resolved. None of these clinical features had a significant value in the univariate and multivariate analyses.

It is possible that the difference observed in OS may be a result of patients with WT KRAS tumours receiving anti-EGFR therapy after the treatment of the study. In our study, 49.8% of patients with WT KRAS tumours received an anti-EGFR agent after bevacizumab treatment. The difference was apparent in the survival curves for patients with WT KRAS tumours who received anti-EGFR, those with WT KRAS tumours who did not receive an anti-EGFR agent and patients with MT KRAS tumours. Patients with WT KRAS tumours who received an anti-EGFR therapy had significantly longer OS than patients with MT KRAS tumours (28.0 versus 20.2 months; HR: 1.68; p = 0.0006), yet there was no significant difference in patients with WT KRAS tumours who were treated with an anti-EGFR versus those who were not (28.0 versus 26.9 months; HR: 1.13; p = 0.5373). Later lines of therapy might influence OS, but response rates and PFS, which are not influenced by second- and third-line therapy, were also better in patients with WT KRAS tumours and may be a more appropriate marker for the success of treatment than OS.

In conclusion, this analysis of the MACRO study highlights the prognostic role of tumour KRAS mutation status in patients receiving chemotherapy in combination with bevacizumab, which is consistent with some literature reports but not others. The reasons for the discrepancy between studies are not yet apparent, and sufficiently powered, prospective studies will be required to answer this question. The importance of KRAS mutation type, as suggested by the RASCAL studies (Andreyev et al 2001; Andreyev et al 1998) and the correlation of KRAS status with microsatellite instability suggested by the PETACC-3 study [24] and other biomarkers such as BRAF, PTEN and PIK3CA [38], remain to be explored.
